# Microbiome-Related Indole and Serotonin Metabolites are Linked to Inflammation and Psychiatric Symptoms in People Living with HIV

**DOI:** 10.1177/11786469221126888

**Published:** 2022-09-27

**Authors:** Nadira Vadaq, Yue Zhang, Elise Meeder, Lisa Van de Wijer, Muhammad Hussein Gasem, Leo AB Joosten, Mihai G Netea, Quirijn de Mast, Vasiliki Matzaraki, Arnt Schellekens, Jingyuan Fu, André JAM van der Ven

**Affiliations:** 1 Department of Internal Medicine, Radboudumc Center for Infectious Diseases, Radboud Institute of Health Science (RIHS), Radboud University Medical Center, Nijmegen, The Netherlands; 2 Center for Tropical and Infectious Diseases (CENTRID), Faculty of Medicine, Diponegoro University, Dr. Kariadi Hospital, Semarang, Indonesia; 3 Department of Genetics, University of Groningen, University Medical Center Groningen, Groningen, The Netherlands; 4 Department of Psychiatry, Radboud University Medical Centre, Nijmegen, The Netherlands; 5 Nijmegen Institute for Scientist-Practitioners in Addiction (NISPA), Nijmegen, The Netherlands; 6 Donders Institute for Brain, Cognition and Behavior, Radboud University, Nijmegen, The Netherlands; 7 Department of Internal Medicine, Faculty of Medicine Diponegoro University-Dr. Kariadi Hospital, Semarang, Indonesia; 8 Department for Immunology and Metabolism, Life and Medical Sciences Institute, University of Bonn, Bonn, Germany; 9 Department of Pediatrics, University of Groningen, University Medical Center Groningen, Groningen, The Netherlands

**Keywords:** Tryptophan metabolism, microbiome, platelet reactivity, inflammation, psychiatric symptoms, HIV

## Abstract

**Background:**

People living with HIV (PLHIV) exhibit dysregulation of tryptophan metabolism. Altered gut microbiome composition in PLHIV might be involved. Mechanistic consequences within the 3 major tryptophan metabolism pathways (serotonin, kynurenine, and indoles), and functional consequences for platelet, immune and behavioral functions are unknown. We investigated plasma tryptophan metabolites, gut microbiome composition, and their association with platelet function, inflammation, and psychiatric symptoms.

**Methods:**

This study included 211 PLHIV on long-term antiretroviral treatment (ART). Plasma tryptophan pathway metabolites were measured using time-of-flight mass spectrometry. Bacterial composition was profiled using metagenomic sequencing. Platelet reactivity and serotonin levels were quantified by flowcytometry and ELISA, respectively. Circulating inflammatory markers were determined using ELISA. Symptoms of depression and impulsivity were measured by DASS-42 and BIS-11 self-report questionnaires, respectively.

**Results:**

Plasma serotonin and indole metabolites were associated with gut bacterial composition. Notably, species enriched in PLHIV were associated with 3-methyldioxyindole. Platelet serotonin concentrations were elevated in PLHIV, without effects on platelet reactivity. Plasma serotonin and indole metabolites were positively associated with plasma IL-10 and TNF-α concentrations. Finally, higher tryptophan, serotonin, and indole metabolites were associated with lower depression and anxiety, whereas higher kynurenine metabolites were associated with increased impulsivity.

**Conclusion:**

Our results suggest that gut bacterial composition and dysbiosis in PLHIV on ART contribute to tryptophan metabolism, which may have clinical consequences for immune function and behavior.

## Graphical Abstract

**Figure:**
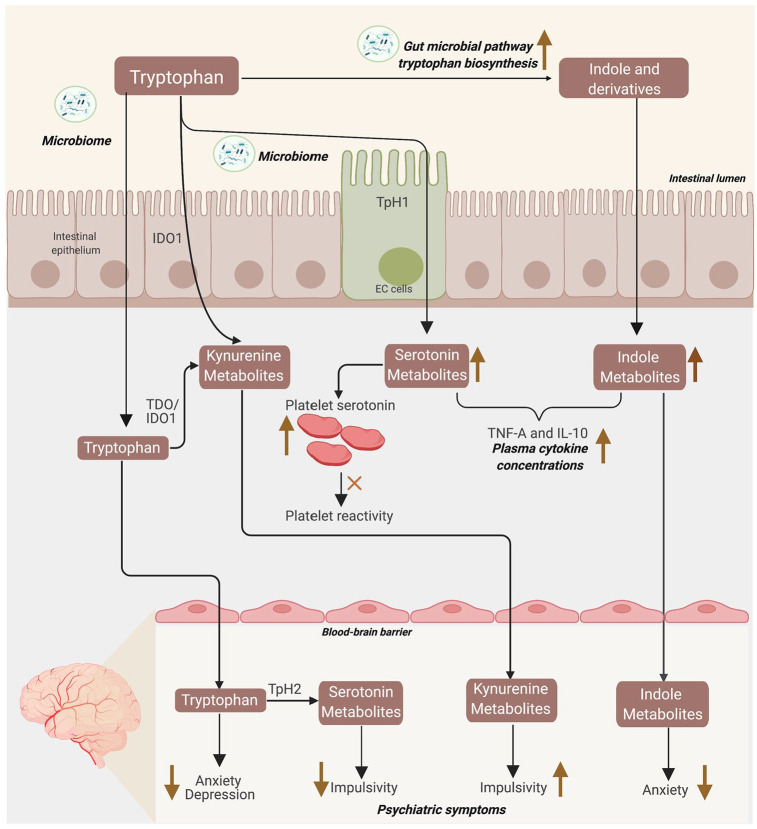
Abbreviations: EC = enterochromaffin cells; TpH1/2 = tryptophan hydroxylase 1/2; IDO1 = indoleamine 2,3-dioxygenase 1; TDO = tryptophan 2,3-dioxygenase; Upper ward facing arrow = positive association; Downward facing arrow = negative association.

## Background

Alteration of tryptophan metabolism has been long known in untreated people living with HIV (PLHIV),^1^ which is only partially normalized by antiretroviral treatment (ART).^2,3^ Tryptophan is a precursor for 3 metabolic pathways: the kynurenine, serotonin, and indole pathway. In humans, tryptophan is mainly metabolized within the kynurenine pathway via the rate-limiting enzyme indoleamine 2,3-dioxygenase 1 (IDO1). In addition, tryptophan is also metabolized into serotonin through the tryptophan hydroxylase 1 (Tph1) enzyme in the gut enterochromaffin (EC) cells. This route is accountable for about 95% of the serotonin in the circulation.^4^

Gut microbiota plays an important role in the regulation of the 3 major pathways of tryptophan metabolism.^5^ Gut microbiota can directly transform tryptophan into indole derivatives,^6,7^ augment the production of kynurenine metabolites through increased expression of gut IDO1,^8,9^ and upregulate serotonin synthesis through the production of secondary bile acids and short-chain fatty acid (SCFAs).^10,11^ PLHIV are known to exhibit gut microbial composition alterations^12-15^ that affect host kynurenine metabolism^13,16,17^ (preprint). However, the influence of gut dysbiosis on serotonin and indole pathways in PLHIV is currently unknown.

Tryptophan and metabolites from its 3 major pathways act as important regulators of various physiological processes in the human body. As a part of the tryptophan metabolism, serotonin is known to regulate a wide range of physiological functions, including supporting platelet reactivity processes.^18-20^ The reason is that after synthesis in the gut, 98% of serotonin is stored in platelet dense granules,^21^ and released into the circulation in the presence of stimuli.^22^ This may lead to platelet activation and aggregation via 5-HT2A receptors.^23^

Next, kynurenine pathway metabolites are known to exhibit immuno-modulatory roles, including in PLHIV.^2,24-28^ In contrast to the kynurenine pathway, the immunological impact of serotonin and indole pathways in PLHIV is less well described.^29^ Serotonin could directly modulate the function of immune cells by binding to serotonin receptors expressed on immune cells,^30,31^ or indirectly through platelet-immune cells interactions after platelet activation. Furthermore, various indole metabolites which are ligands of aryl hydrocarbon receptor (AhR), are known to have a beneficial effect on the host intestinal immunity.^6,32,33^

Tryptophan metabolism might affect the central nervous system (CNS) via all 3 major pathways. Firstly, tryptophan is the sole precursor of neurotransmitter serotonin via the rate-limiting enzyme Tph2 in CNS.^34,35^ Secondly, kynurenine is able to pass the blood-brain barrier (BBB) and associated with clinical symptoms in psychiatric patients.^36^ Thirdly, indoles of bacterial origin can be absorbed into the circulation, cross the BBB and exhibit neuroprotective effects through AhR signaling.^37^ Therefore, alterations in tryptophan metabolism may affect the blood-brain barrier and thereby relate to psychiatric symptomatology in PLHIV. Indeed, several studies in PLHIV have related the degradation of tryptophan into kynurenine to symptoms of depression.^38,39^ Yet, a comprehensive assessment of the relation between the 3 major tryptophan metabolism pathways and psychiatric symptomatology in PLHIV is lacking.

Given that HIV infection is characterized by dysregulation in tryptophan metabolism, which partly has been associated with gut dysbiosis, we aimed to assess whether serotonin, kynurenine, and indole metabolism are associated with alterations of gut bacterial composition in PLHIV using long-term ART. Next, to further explore the functional consequences of tryptophan metabolism, we assessed relationships between tryptophan metabolism and platelet reactivity, circulating inflammatory markers, and symptoms of depression, anxiety, stress, and impulsivity in PLHIV using long-term ART.

## Methods

### Study population

#### Design

We assessed cross-sectionally tryptophan metabolism, bacterial composition, and clinical phenotypes related to immune function and psychiatric symptoms in a cohort of virally suppressed PLHIV (200 HIV cohort). For platelet serotonin measurements, we used 2 independent PLHIV cohorts: (1) the 200-HIV cohort and (2) the Art-NeCo cohort.

#### 200 HIV cohort

The 200-HIV cohort was part of the Human Functional Genomic Project (HFGP; www.humanfunctionalgenomics.org). A total of 211 PLHIV were recruited between December 2015 and February 2017 in the 200-HIV cohort.^40,41^ The inclusion criteria of this study include a minimum age of 18 years old, receiving ART more than 6 months, and HIV-RNA levels <200 copies/mL. The exclusion criteria were acute or opportunistic infections or taking antibiotics less than a month prior to study enrollment and having an active hepatitis B/C infection. Data on sexual behavior, such as men who have sex with men (MSM) or men who have sex with women (MSW) were recorded.

#### Art-NeCo cohort

Validation of increased platelet serotonin concentration in PLHIV was investigated in participants from the Art-NeCo cohort, who visited Radboudumc between February 2014 and September 2014. PLHIV were eligible for inclusion if they had received ART for at least 1 year, HIV-RNA levels <20 copies/mL, and no signs of opportunistic infections or malignancies. For this cohort, we included 56 viral suppressed PLHIV.^42,43^

#### Study approval

The 200HIV and Art-NeCo studies were conducted in accordance with the Declaration of Helsinki after approval by the Medical Ethical Review Committee region Arnhem-Nijmegen, The Netherlands (42561.091.122 and 2011/267 respectively).

### Plasma metabolomics measurements

Plasma metabolomics measurements were performed using flow injection electrospray–time-of-flight mass spectrometry by General Metabolics (Massachusetts, United States), according to the previously described methodology.^44,45^ There were no dietary restrictions prior to blood collection. Venous blood sample was collected for each participant in EDTA BD Vacutainer® tubes between 8 and 11 am and stored at −80°C prior to measurements. Metabolites were identified according to the mass-to-charge ratio and annotated using the Kyoto Encyclopedia of Genes and Genomes (KEGG) database^46^ and the Human Metabolome Database (HMDB).^47^

### Metagenomic data generation and profiling

Metagenomic sequencing and data processing was described before^17^ (preprint). Fecal DNA isolation was performed using the QIAamp Fast DNA Stool Mini Kit (Qiagen; cat. 51604), and then fecal DNA was sent to Novogene to conduct library preparation and perform whole genome shotgun sequencing on the Illumina HiSeq platform. Low quality reads and reads belonging to human genome were removed by mapping the data to the human reference genome (version NCBI37) with kneaddata (v0.7.4).

Microbial taxonomic and functional profiles were determined using Metaphlan3 (v3.0.7)^37^ and HUMAnN3 (v3.0.0.alpha.3).^37^ The reads identified by MetaPhlAn3 were mapped to species-specific pangenomes with UniRef90 annotations, and the MetaPhlAn3-unclassified reads were translated and aligned to a protein database. Bacteria/pathways present in <20% of the samples from one cohort were discarded.

The differential abundance of bacterial species between 200HIV cohort and HC (DMP cohort), independent of sexual behavior and other confounders, has been reported^17^ (preprint). In summary, there were increased abundances of 57 bacterial species, especially *Prevotella* and *Prevotellaceae* genera, and depletion of 19 bacterial species, such as *Barnesiella*, *Alistipes*, and *Bacteroides* genera, in PLHIV compared to HC.

### Platelet serotonin concentration measurements

Platelet serotonin concentration in the 200HIV cohort (n = 211PLHIV) was determined using ELISA (IBL-International, Hamburg, Germany), according to the manufacturer’s protocols. Whole blood samples were collected using the CTAD tube and immediately centrifuged at 150*g* for 10 minutes without a break to obtain platelet-rich plasma (PRP). Platelet count in PRP was determined by Sysmex XN-450 automated hematology analyzer (Sysmex Corporation, Kobe, Japan). To obtain the platelet pellets, 200 µL of PRP was added to 800 µL of physiological saline prior to centrifugation at 4500*g* for 10 minutes at 4°C. Next, 200 µL of double-distilled water was added to the pellets before being stored at 80°C. Thawed samples were centrifuged at 10 000*g* for 2 minutes at room temperature. Then, 20 μL of the supernatant was added into a culture tube, followed by 100 μL buffer solution. Incubation was done for 15 minutes at 37°C water bath after the addition of 25 μL acylation reagents. After centrifugation for 10 minutes at 1500*g*, platelet serotonin concentration was measured in 200 μL supernatant within 1 hour. For comparison, we simultaneously measured the platelet serotonin in 56 samples from healthy volunteers. Sample collection and processing was done simultaneously and in the same way as in the 200-HIV cohort by the same personnel.^40,41^

In Art-NeCo cohort, blood samples (n = 56 PLHIV) were collected using CTAD containing vacutainers. Blood samples were directly centrifuged at 150*g* for 10 minutes without a break to obtain PRP, which was pipetted off and further processed by centrifuging at 1350*g* for 15 minutes to a platelet pellet with a known number of platelets. Platelet pellets were lysed using perchloric acid and stored at –80°C until analysis by Department of Clinical Chemistry and Hematology of the University Medical Center Utrecht. Intraplatelet serotonin concentration was determined in the supernatant of the platelet pellets, which was obtained after centrifuging the thawed samples for 10 minutes at 2000*g*. The supernatant was aspired and hydrochloric acid was added before reading the fluorescence of serotonin after exciting at 310 nm and registering at 510 nm by a Fluorescence Spectrophotometer (F-7000, Hitachi High Technologies, Schaumburg, IL, USA). This assay had a lower detection limit of 170 nmol/10^11^ platelets and an intra-assay CV below 10% as described earlier.^48^ For comparison, we measured the platelet serotonin in 11 samples from healthy volunteers. Sample collection and processing was done simultaneously and in the same way as in the Art-NeCo cohort by the same personnel.^42,43^ For the 200-HIV and Art-Neco Cohort, the platelet serotonin concentration was adjusted to platelet count and referred to as serotonin (ng/10^9^ platelets).

### Platelet reactivity assay

Whole blood samples were collected inside a 3.2% sodium citrate tube (Becton Dickinson, Franklin Lakes, NJ, USA) and processed between 1 and 3 hours after blood collection using flow cytometry-based assay as described before.^49^ Platelet reactivity was assessed under basal (unstimulated) conditions and after stimulation with adenosine diphosphate (ADP; 1.2 and 125uM; Sigma-Aldrich, Zwijndrecht, The Netherlands), cross-linked collagen-related-peptide (CRP-XL; a kind gift from Prof. Farndale, Cambridge, UK), and co-stimulation between ADP with 50 uM serotonin (Sigma-Aldrich, Zwijndrecht, The Netherlands). Platelet reactivity was defined by median fluorescence intensity (MFI) of α-granule protein P-selectin (Biolegend, San Diego, CA, USA) expression and fibrinogen (DAKO, Santa Clara, CA) binding to the activated integrin αIIbβ3. All data were extracted using Kaluza 2.1 software (Beckman Coulter, France) and normalized against quality controls to ensure measurement stability.

### Circulating inflammatory markers

The circulating inflammatory markers were measured in EDTA plasma samples of PLHIV from the 200-HIV cohort using ELISA according to the manufacturer’s instructions. Absolute concentrations of interleukins (IL-10, IL-6, IL-1β, IL-1RA, and tumor necrosis factor-alpha [TNF-α]) were measured using SimplePlex Cartridge (Protein Simple, San Jose, CA, USA). Markers of persistent inflammation (high-sensitivity C-reactive protein (hsCRP), α-1 anti-trypsin (AAT), soluble (s) CD14, and sCD163, IL-18, IL-18 BP, and marker of intestinal barrier dysfunction (intestinal fatty acid-binding protein [I-FABP]) were measured using ELISA (Duoset or Quantikine, R&D Systems).

### Psychiatric measurements

Symptoms of depression, anxiety and stress, and impulsivity levels were measured in PLHIV of the 200-HIV cohort using self-report questionnaires; the Depression Anxiety Stress Scale 42 (DASS-42) and the Barratt Impulsiveness Scale (BIS-11), respectively.^50,51^ An elaborate description of these questionnaires has been published elsewhere.^45^ Psychiatric symptom levels were expressed as a total score of DASS-42 and its subscores (depression, anxiety, and stress), and a total score of BIS-11 and its subscores (attentional, motor impulsivity, and non-planning impulsivity). DASS-42 and BIS-11 have high validity and internal consistency and both have been used in PLHIV populations in previous literature.^52-54^

### Statistical analysis

A detailed description of the statistical methods can be found in the Supplemental Methods. The descriptive statistics of the population were described as medians and interquartile ranges for continuous variables and as numbers and percentages for categorical variables. The number of participants with available data across parameters measured in each cohort is presented in Supplemental Table 3. Spearman’s correlation was used for univariate correlation analyses and a multivariate linear regression model was used to correct for age and sex unless otherwise stated. Prior to association analysis with the parametric approach, data were inverse ranked transformed to follow a standard normal distribution. Correction for multiple testing procedures was performed using the false discovery rate (FDR) method. Statistical analysis and visualization were performed using R (version 4.0.1; Vienna, Austria).

## Results

Baseline characteristics of the 200-HIV cohort have been described previously.^40,41^ Included PLHIV were predominantly males (193/211 [91.5%]), with a median age of 52.5 years (IQR 46.2-59.4). Subjects had been treated for a median of 6.6 years [**TS: Please change 6:6 years to 6.6 years.]** (IQR 4.2-11.9) and had substantial immune recovery (CD4 count median 660 cells/mL [IQR 480-810]) (Supplemental Table 1).

### Tryptophan metabolism in PLHIV

**Figure 1.**
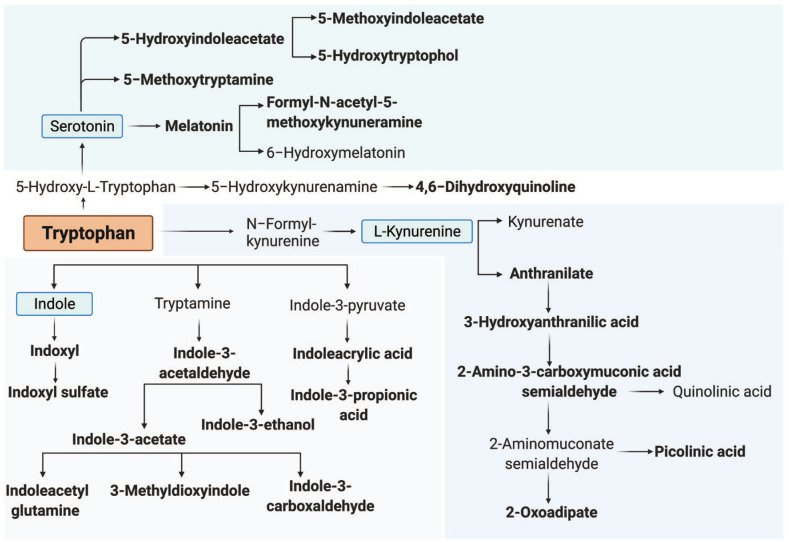
Overview of the tryptophan metabolism pathway. Overview of tryptophan metabolism pathway and its 3 sub-pathways: serotonin, kynurenine, and indole pathway. Metabolites that were measured in the 200 HIV cohort are shown in bold.

**Figure 2.**
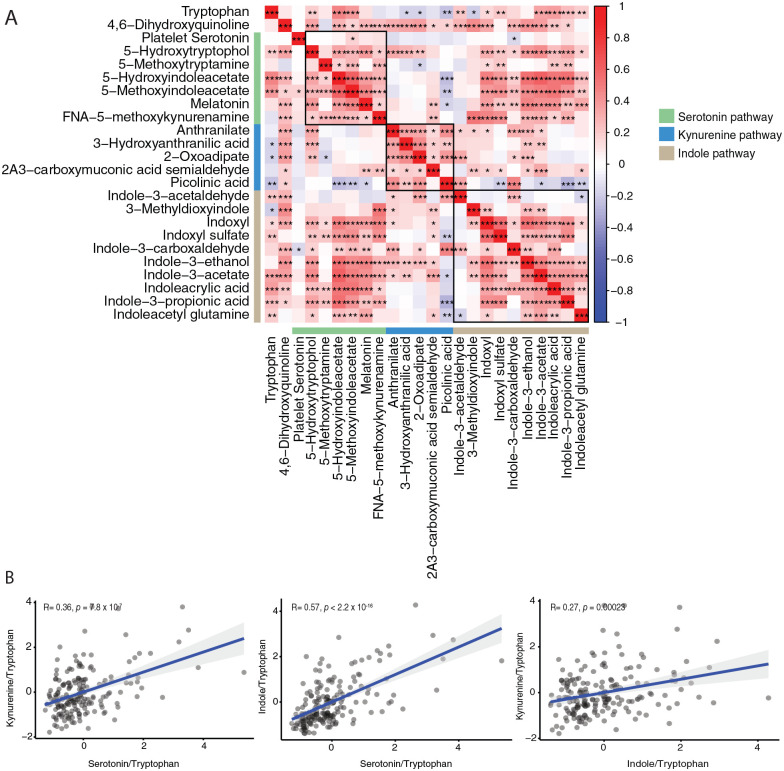
Association between serotonin, kynurenine, and indole pathway metabolites: (A) correlation among metabolites of tryptophan metabolism pathway in PLHIV. Red and blue color indicates a positive and negative correlation respectively. Green, blue, and brown sidebar represents metabolites from serotonin, kynurenine, and indole pathway, respectively. Asterisk (*) indicates statistical significance after FDR multiple testing correction at *P* < .05 (*), *P* < .001 (**), and *P* < .0001 (***) using spearman’s rank correlation and (B) correlation between the fixed composite value of serotonin, kynurenine, and indole pathways per tryptophan. *P*-values and Spearman’s coefficients (*R*) are shown in the scatter plot. Abbreviation: 2A3-carboxymuconic acid acetaldehyde, 2-Amino-3-carboxymuconic acid acetaldehyde; FNA-5-methoxykynurenamine, formyl-N-acetyl-5-methoxykynurenamine.

We first determined the interrelation of metabolites of tryptophan metabolism pathways (Figure 1). In general, strong significant associations were found among metabolites of the tryptophan metabolism pathway, particularly among downstream metabolites of the serotonin, kynurenine, and indole pathways (Figure 2A) as expected. Next, we explored the activity of serotonin, kynurenine, and indole pathway metabolism by correlating the composite value of the corresponding metabolites in each pathway to tryptophan ratio. Overall, we found a positive association between tryptophan metabolism and serotonin, kynurenine, and indole pathways (*P* < .05) (Figure 2B). The strongest correlation was observed between the serotonin and indole pathways (*P* < 2.2 × 10^−16^, *R* = .57).

### Gut microbiota association to tryptophan metabolism

**Figure 3.**
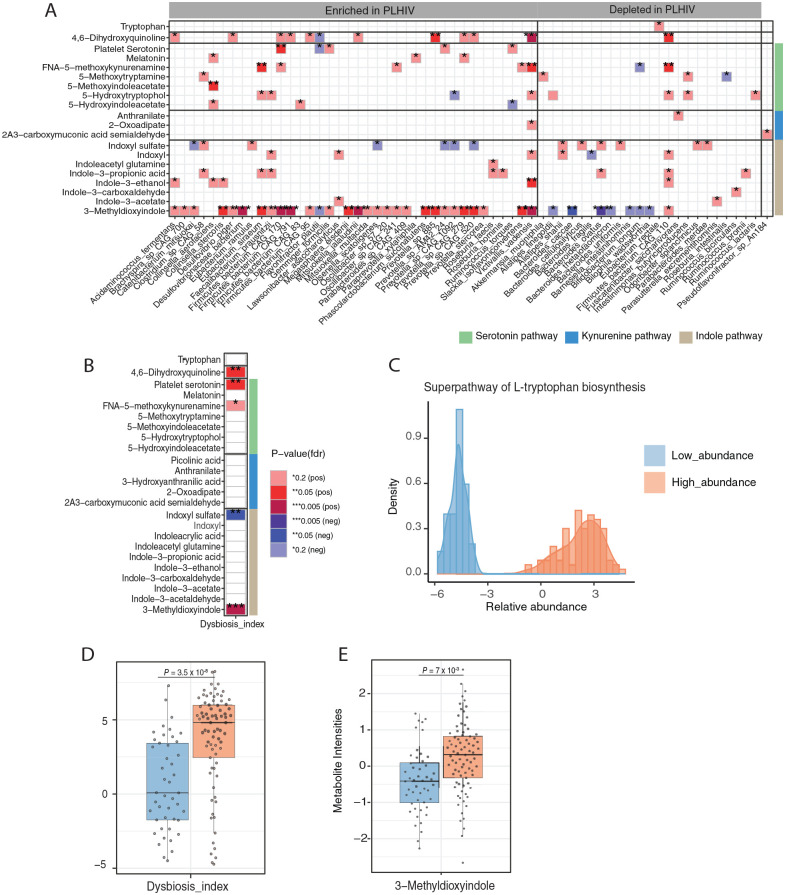
Association between plasma metabolites of tryptophan metabolism pathway and gut bacterial composition: (A) heatmap presenting significant associations (FDR < 0.2) between metabolites from the tryptophan metabolism pathway and relative abundance of gut bacterial species and (B) dysbiosis index in PLHIV. Green, blue, and brown sidebar represent metabolites from serotonin, kynurenine, and indole pathway respectively, (C) histogram of relative abundance of bacteria belonging to the “Superpathway L-Tryptophan biosynthesis” as measured in PLHIV, (D) box plots depicting the dysbiosis index, and (E) relative abundance of 3-methyldioxyindole in PLHIV stratified by high (n = 81) and low abundance group (n = 48). In each box plot, the in-box line defines the median value, hinges depict 25th and 75th percentiles and whiskers extend to ±1.5 interquartile ranges; each dot indicates an individual. All values were inversed ranked transformed. The analysis was performed using a linear regression model using age, sex, and read counts. Abbreviation: 2A3-carboxymuconic acid acetaldehyde, 2-Amino-3-carboxymuconic acid acetaldehyde; FNA-5-methoxykynurenamine, formyl-N-acetyl-5-methoxykynurenamine.

Given that tryptophan metabolism is one of the well-known metabolic pathways involved in the host-microbiome interaction, we assessed the association between the relative abundance of gut bacterial species and metabolites of tryptophan metabolism in PLHIV. Out of 99 gut bacterial species which were detected in ⩾20% of samples, 62 species (62.5%) were associated with at least 1 metabolite of the tryptophan metabolism pathway (FDR < 0.2) after correction for age, sex and read counts. Notably, most gut bacterial species that were enriched in PLHIV were associated with 3-methyldioxyindole, an indole pathway metabolite (Figure 3A, Supplemental Table 4). Significant associations were also found between bacterial species with the metabolites of the serotonin pathway and 4,6-dihydroxyquinoline. On the contrary, there was hardly any association observed between bacterial species and metabolites of the kynurenine pathway. Dysbiosis index was positively associated with 3-methyldioxyindole, 4,6-dihydroxyquinoline, and platelet serotonin concentrations (Figure 3B, Supplemental Table 5), and negatively associated with indoxyl sulfate (FDR < 0.05).

Next, we focused on gut microbiota with a known function in tryptophan metabolism. We observed a clear bimodal distribution of the bacterial metabolic pathway “Superpathway of L-tryptophan biosynthesis” in PLHIV, with one group having a high abundance (n = 81), and the other a low abundance (n = 48) (Figure 3C). The high abundance group had a significantly higher dysbiosis index compared to the low abundance group (*P* = 3.5 × 10^−8^) (Figure 3D). Furthermore, we looked at the possible difference in the host-tryptophan metabolic profile between the high and low abundance groups. As expected, the high abundance group had significantly higher 3-methyldioxyindole (*P* = .007) (Figure 3E) and a trend of lower tryptophan compared to the low abundance group (Supplemental Figure 2). The high and low abundance groups did not differ in platelet serotonin concentrations or other metabolites of the tryptophan metabolism (Supplemental Figure 1).

Given that sexual behavior is an important determinant affecting gut bacterial composition in PLHIV (preprint),^17,55^ we also tested whether sexual behavior had an effect on the association between gut bacterial composition and metabolites of the tryptophan metabolism pathway. In this study, 70% and 17.5% of PLHIV were men having sex with men (MSM) and men having sex with women (MSW) respectively. Upon conducting analysis only for men and performing correction for sexual orientation (MSM/MSW), associations between gut bacterial species with metabolites of serotonin and indole pathways remained significant (FDR < 0.2). These results suggested that the influence of gut bacterial composition was independent of sexual behavior in PLHIV (Supplemental Figure 2, Supplemental Table 6).

### Increased platelet serotonin concentration in virally suppressed PLHIV and its relation with platelet reactivity

Given the significant association between gut bacterial composition with the serotonin pathway in PLHIV, we investigated the possible difference in platelet serotonin concentration between PLHIV and HC. Fifty-six HC were enrolled simultaneously with PLHIV of 200HIV cohort, and platelet serotonin concentration was determined in all individuals. Detailed characteristics of the HC have been described previously.^40,41^ In summary, PLHIV were older (median [IQR] 52.5 [46.2-59.4] years) compared to HC participants (median [IQR] 30 [25.1-52.2] years) (*P* < .05). PLHIV had significantly more male participants (males 193/211 [91.5%]) than HC (males 34/56 [60.7%]) (*P* < .05).

**Figure 4.**
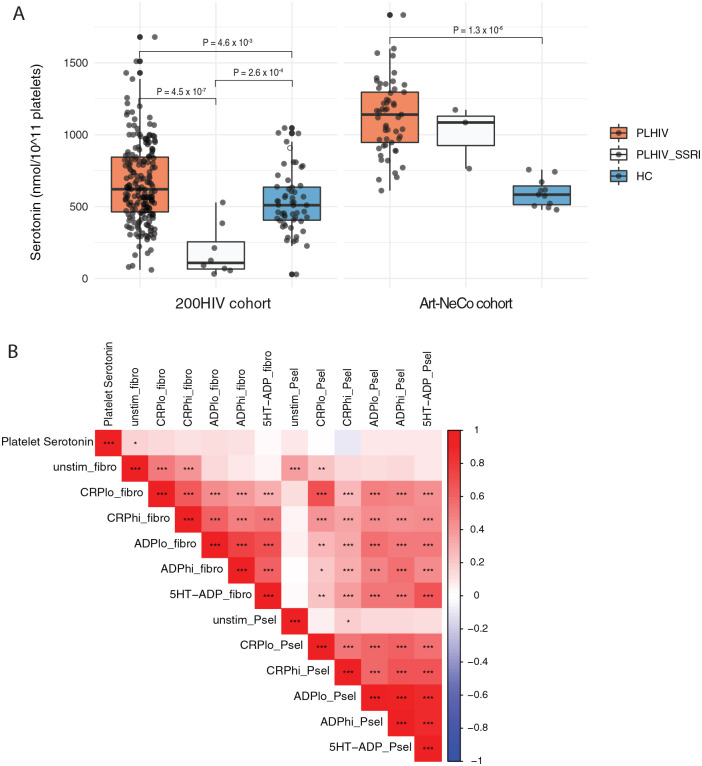
Comparison of platelet serotonin concentration between PLHIV and healthy controls: (A) comparison of platelet serotonin concentration between PLHIV non-SSRI users, PLHIV-SSRI users, and healthy controls in 200HIV cohort (left) and Art-NeCo cohort (right). The analysis was performed by linear regression model using age and sex as covariates. In each box plot, the in-box line defines the median value, hinges depict 25th and 75th percentiles and whiskers extend to ±1.5 interquartile ranges; each dot indicates an individual participant and (B) association between platelet serotonin concentration and platelet reactivity parameters in PLHIV of 200HIV cohort. Red and blue cells indicate a positive and negative correlation, respectively. Asterisk (*) indicates statistical significance after FDR multiple testing correction at *P* < .05 (*), *P* < .001 (**), and *P* < .0001 (***) using spearman’s rank correlation. Abbreviations: Unstimulated P-selectin expression (unstim_Psel) and fibrinogen binding (unstim_fibro); low and high dose of ADP-stimulated P-selectin expression (ADPlo_Psel and ADPhi_Psel) and fibrinogen binding (ADPlo_fibro and ADPhi_fibro); low and high dose of CRP-XL-stimulated P-selectin expression (CRPlo_Psel and CRPhi_Psel) and fibrinogen binding (CRPlo_Fibro and CRPhi_Fibro); low dose of ADP and serotonin-stimulated P-selectin expression (5HT-ADP_Psel) and fibrinogen binding (5HT-ADP_fibro).

PLHIV had a significantly increased platelet serotonin concentration compared to HC, with median values of 620.9 (IQR 462.88-843.92) nmol/10^11^ platelets and 509.42 (IQR 405.23-635.26) nmol/10^11^ platelets, respectively (*P*-value = .00529) (Figure 4A). Platelet serotonin concentrations of selective serotonin reuptake inhibitors (SSRI) users (median [IQR] 108.07 [65.4-254.69] nmol/10^11^ platelets) were significantly lower compared to non-SSRI users (*P* = 4.5 × 10^−7^) and HC (*P* = .000492).

To validate our results, we compared platelet serotonin concentration in a second independent PLHIV cohort (Art-NeCo cohort, n = 56) and 11 healthy individuals. The 56 PLHIV and 11 HC had a median age of 52 (IQR 46.7-61) and 27 years (IQR 25.5-30), respectively. Enrolled PLHIV and HC were predominantly males (89% [50/56] and 73% [8/11]). All PLHIV were on stable ART for a median of 10.1 years (IQR 5-15.5), with a median CD4+ count of 655 cells/mL (IQR 473-818). Consistent with our previous findings, platelet serotonin concentration was higher in PLHIV (median [IQR] 1140 [946-1296] nmol/10^11^ of platelets) compared to HC (median (IQR) = 583 (513-643.5) nmol/10^11^ platelets) (*P* = 1.3 × 10^−6^) (Figure 4A). SSRI treatment (n = 3) did not affect platelet serotonin concentrations in this sample.

Next, we looked at the association between platelet reactivity parameters and platelet serotonin concentration in PLHIV of the 200HIV cohort. No significant associations were found between platelet serotonin concentration and platelet reactivity parameters (Figure 4B). Although underpowered (n = 8), platelet reactivity parameters also did not differ between SSRI users compared to non-SSRI users.

### Association between plasma tryptophan metabolism pathway and circulating inflammatory markers

**Figure 5.**
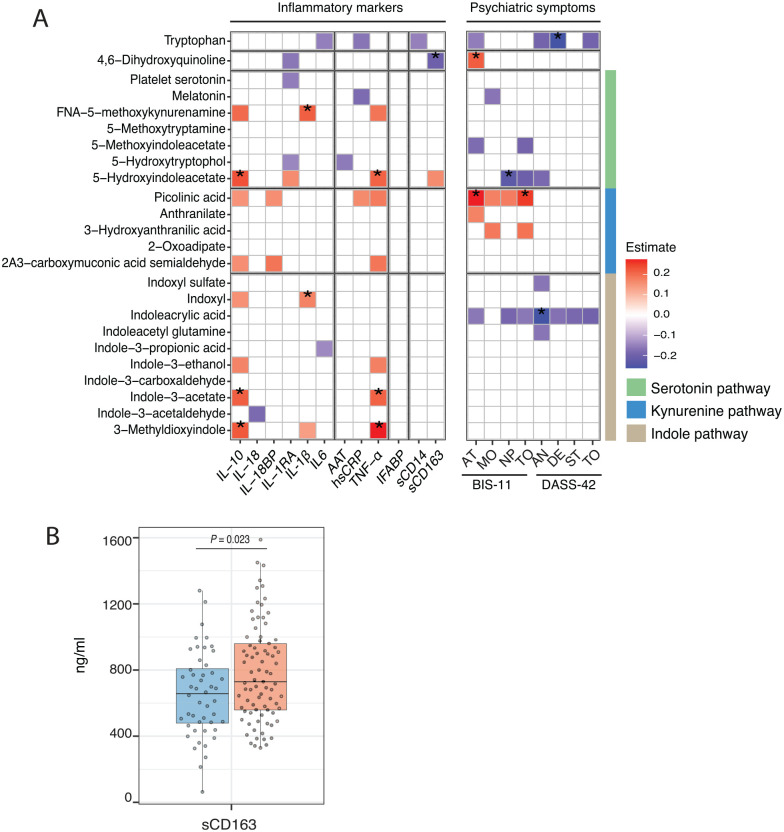
Association between plasma metabolites of tryptophan metabolism and inflammatory markers and psychiatric symptoms: (A) tryptophan metabolism association to inflammatory markers and psychiatric symptoms. (Left) Heatmap presenting the estimate of significant association (*P* < .05) between tryptophan metabolism pathway and plasma inflammatory markers in PLHIV. (Right) Heatmap presenting the estimate of significant associations (*P* < .05) between tryptophan metabolism pathway and DASS-42 and BIS-11 subscores in PLHIV. The analysis was performed using a linear regression model using age and sex as covariates. Asterisk (*) indicates statistical significance at FDR < 0.2. Green, blue, and brown sidebar represent metabolites from serotonin, kynurenine, and indole pathway respectively and (B) box plots depicting the difference in sCD163 concentration between the high (n = 81) and low abundance group (n = 48) of “Superpathway L-Tryptophan biosynthesis” microbial pathway in PLHIV. The in-box line defines the median value, hinges depict 25th and 75th percentiles and whiskers extend to ±1.5 interquartile ranges; each dot indicates an individual participant. Abbreviations: 2A3-carboxymuconic acid acetaldehyde, 2-Amino-3-carboxymuconic acid acetaldehyde; AN, anxiety; AT, attentional impulsivity; DE, depression; FNA-5-methoxykynurenamine, formyl-N-acetyl-5-methoxykynurenamine; MO, motor impulsivity; NP, non planning impulsivity; ST, stress; TO, total DASS-42 or BIS-11 score.

To explore possible immunological consequences of alterations in tryptophan metabolism in PLHIV, we related tryptophan and its 3 major pathways with 12 circulating inflammatory markers (IL-18, IL-18 BP, IL-10, IL-1RA, IL-1β, IL-6, TNF-α, hsCRP, AAT, I-FABP, sCD14, and sCD163) in PLHIV. Significant positive associations were mainly found between metabolites of serotonin (5-hydroxyindoleacetate), and indole (3-methyldioxyindole, indole-3-acetate) pathways with both anti-inflammatory IL-10 (*P*_3-methyldioxyindole_ = 0.004, Estimate_3-methyldioxyindole_ = 0.23; *P*_indole-3-acetate_ = 0.004, Estimate_indole-3-acetate_ = 0.22; *P*_5-hydroxyindoleacetate_ = 0.001, Estimate_5-hydroxyindoleacetate_ = 0.23; all FDR < 0.2) and pro-inflammatory TNF-α (*P*_3-methyldioxyindole_ = 0.0004, Estimate_3-methyldioxyindole_ = 0.28; *P*_indole-3-acetate_ = 0.004, Estimate_indole-3-acetate_ = 0.21; *P*_5-hydroxyindoleacetate_ = 0.004, Estimate_5-hydroxyindoleacetate_ = 0.21; all FDR < 0.2) (Figure 5A, Supplemental Table 7). Furthermore, IL-1β concentrations were positively associated with formyl-N-acetyl-5-methoxykynurenamine (*P* = .0008, Estimate = 0.22; FDR < 0.2) and indoxyl (*P* = .006, Estimate = 0.17; FDR < 0.2). To note, plasma metabolites of the tryptophan metabolism pathway did not show significant associations with IFABP, a marker for microbial translocation. Interestingly, 4,6,dihydroxyquinoline was negatively associated with the absolute concentration of sCD163, a marker of monocyte activation (*P* = .005, Estimate = −0.2; FDR < 0.2) (Figure 5A, Supplemental Table 7).

Next, we assessed the possible difference in the plasma inflammatory markers between the high and the low abundance groups of gut bacterial pathway tryptophan biosynthesis. The high abundance group had significantly higher sCD163 than the low abundance group (*P* = .023); no differences were found for other inflammatory markers (Figure 5B).

### Association between plasma tryptophan metabolism and psychiatric symptoms

Given the possible link of tryptophan metabolism to psychiatric conditions,^38,39^ we explored the clinical consequences of alterations in tryptophan metabolism in PLHIV. Higher tryptophan was significantly associated with lower depression levels (DASS-42; *P* = .0009, Estimate = −0.23); higher 5-hydroxyindoleacetate with lower non-planning impulsivity levels (BIS-11; *P* = .002, Estimate = −0.22); and higher indoleacrylic acid with lower anxiety levels (DASS-42; *P* = .001, Estimate = −0.23) (FDR < 0.2). Higher picolinic acid, a downstream metabolite of the kynurenine pathway, was associated with higher attentional impulsivity levels (BIS-11; *P* = .0002, Estimate = 0.27) and total BIS-11 scores (*P* = .0008, Estimate = 0.25) (FDR < 0.2) (Figure 5A, Supplemental Table 8). No associations were found between psychiatry symptoms and gut bacterial pathway tryptophan biosynthesis.

## Discussion

The main finding of this study is the positive relation between HIV-associated microbial alterations and plasma concentrations of metabolites of the serotonin and indole pathways of tryptophan metabolism in virally suppressed PLHIV on ART. Notably, bacterial species enriched in PLHIV were associated with 3-methyldioxyindole levels. In addition, this study unravels the functional consequences of alterations in tryptophan metabolism in PLHIV on markers of systemic inflammation and psychiatric symptoms. Plasma metabolites of indole and serotonin pathways were associated with anti-inflammatory IL-10, and pro-inflammatory TNF-α levels. Higher tryptophan and indoleacrylic acid levels were associated with decreased levels of depression and anxiety respectively, while a higher level of the kynurenine metabolite picolinic acid was associated with increased impulsivity.

Enriched gut bacterial species in PLHIV were mostly associated with plasma metabolites of the indole pathway, particularly with 3-methyldioxyindole, suggesting the upregulation of indole-containing metabolites production by gut microbiota in PLHIV. Given that the production of indoles from tryptophan depends heavily on the gut microbial community,^6,7,32^ we observed significantly higher plasma 3-methyldioxyindole and a trend for lower tryptophan in PLHIV with a high abundance of tryptophan catabolizing bacteria compared to those with low abundance. Furthermore, various gut bacterial species, including SCFAs producing bacteria, such as *Firmicutes* and *Eubacterium* species, and dysbiosis index were positively associated with downstream metabolites of the serotonin pathway, suggesting a significant influence of gut microbiota dysbiosis on serotonin pathway in PLHIV.

We also observed increased platelet serotonin levels in PLHIV, compared to healthy individuals, which pinpoints the positive relation between gut microbiota and the serotonin pathway. In agreement with our findings, a 6-month course of probiotics has been shown to significantly increase serum serotonin in PLHIV on ART.^56^ As we found no difference in plasma metabolites of the serotonin pathway between the high and low abundance groups of gut bacterial pathway tryptophan biosynthesis, it is unlikely that the increase in platelet serotonin levels directly results from increased production by the gut microbiota. Instead, we assume that the gut microbiome may indirectly modulate these processes. Previous studies in mouse models have shown an upregulation of TPH1 in gut EC cells through productions of secondary bile acids and SCFAs.^10,11^ In the present study, we hardly found any significant association between gut bacterial composition and metabolites of the tryptophan pathway in PLHIV. In agreement with our findings, a study in 15 PLHIV on ART revealed that 8 weeks of probiotic supplementation had no effect on kynurenine and the kynurenine tryptophan ratio.^57^

Our study found no relation between platelet serotonin and platelet reactivity, suggesting that platelets act as a serotonin storage site, without functional consequences for platelets. This may be explained by decreased 5-HT2a receptor expression on the platelet surfaces of these individuals, as prior findings show a reciprocal relation between platelet serotonin concentration and platelet 5-HT2A receptor expression.^58^ This may be a protective mechanism as high concentrations of plasma serotonin caused by increased platelet activation may have a serious hemodynamic impact.^59^

Serotonin is known to regulate various immune cell functions and differentiation.^31,60-62^ In addition, numerous studies have described the impact of indole metabolites on inflammation and the mucosal barrier.^6,32,33,63^ Of note, we observed positive associations between metabolites of the serotonin (5-hydroxyindoleacetate) and indole (3-methyldioxyindole, indole-3-acetate) pathways with pro-inflammatory TNF-α and anti-inflammatory IL-10 concentrations. In line with our findings, a study in human intestinal organoids and intestinal epithelial cell lines revealed the role of microbiome-derived-indole metabolites on induction of IL-10R1 expression.^64^ While no study has reported the immunological effects of 3-methyldioxyindole, its upstream metabolites, 3-methylindole/skatole, have been associated with disrupted intestinal homeostasis and intestinal epithelial cell death.^65^ In contrast to our results, a previous study conducted in 49 PLHIV on ART reported no association between indole-3-acetic acid and 5-hydroxyindoleacetate with markers of inflammation.^66^

Apart from the serotonin and indole pathway, upregulation of the kynurenine pathway has been associated with intestinal and systemic inflammation in PLHIV.^2,25,29,67^ In this study, downstream metabolites of the kynurenine pathway were not associated with plasma inflammatory markers. However, it is important to note that this study did not measure kynurenine pathway metabolites with known immune-modulatory effects, such as kynurenine and kynurenic acid. Overall, these findings suggest a positive influence of serotonin and indole pathway metabolites on plasma cytokine concentrations in PLHIV. Future studies should investigate the net effect of these metabolites on the balance of pro-and anti-inflammatory cytokine concentrations in PLHIV.

Furthermore, we identified that higher plasma tryptophan was associated with lower levels of depression, anxiety, and stress in PLHIV. This finding is in line with previous studies in PLHIV that report significant relations between symptoms of depression and disturbed tryptophan metabolism.^39,68^ In addition, we found significant associations between higher anxiety levels and lower indoleacrylic acid and a trend for lower indoxyl sulfate, both indole products of the microbiome. The role of the microbiome and fear extension learning deficits and its relation to anxiety disorders was recently demonstrated in a mouse study,^69^ in which the role of the indole metabolite indoxyl sulfate was also highlighted. In line with our findings, indoleacrylic acid and its downstream metabolite have been shown to exhibit neuroprotective effects.^37,70^

Moreover, we found a positive association between picolinic acid, a product of the kynurenine pathway, with both attentional impulsivity and the total BIS-11 score. Picolinic acid is known for its neuroprotective role, while it is also known to have antiviral, antifungal, anti-tumor and cell growth regulatory properties.^71-73^ High picolinic acid concentrations were found in patients suffering from bipolar disorder,^74^ which might relate to impulsivity during manic periods. Picolinic acid is also known to have antiviral effects on HIV and other viruses, such as herpes simplex,^73^ which are known to contribute to CNS inflammation in PLHIV. Our data do not support previous findings of lower levels of picolinic acid in depressed patients,^75^ including in PLHIV.^39^ Altogether, these findings suggest a protective role of tryptophan, plasma metabolites of serotonin, and indoles on psychiatric symptoms in PLHIV, while kynurenine metabolites showed an opposite effect. Our findings also corroborate previous reports of a disturbance of the microbiome-gut-brain axis in PLHIV (reviewed by^76^).

The present study bears some limitations. First, while we have data on platelet serotonin in both PLHIV and HC, we have no comparable data on tryptophan metabolism in healthy individuals, which makes it difficult to conclude whether the effects are HIV-specific. Second, we did not cover a complete set of tryptophan metabolism metabolites, especially in the kynurenine pathway. This may limit possible findings on the functional consequences of other bioactive metabolites of tryptophan metabolism in PLHIV. Third, we did not use a stringent multiple testing correction in the analysis between plasma metabolites of tryptophan metabolism with plasma markers and psychiatric symptoms due to the exploratory nature of the analyses. Next, being a cross-sectional study, no conclusions on causality can be drawn, and further validation in prospective experimental studies is needed. Lastly, the small number of female participants in this study may limit the interpretation of the findings in the general HIV population.

The present study demonstrates for the first time gut bacterial influence on serotonin and indole metabolism in well-treated PLHIV. Plasma tryptophan metabolites may exhibit distinct effects on inflammation and psychiatric symptoms. Our data underscore the complex regulation and interconnection of tryptophan metabolic pathways with possible various phenotypic consequences in PLHIV. Modulation of enzymes, metabolites, or receptors involved in multi-faceted tryptophan metabolism or development of next-generation probiotics should be carefully formulated to achieve considerate benefits in controlling persistent inflammation and psychiatric symptoms in PLHIV in which intestinal dysbiosis is involved.

## Supplemental Material

sj-docx-1-try-10.1177_11786469221126888 – Supplemental material for Microbiome-Related Indole and Serotonin Metabolites are Linked to Inflammation and Psychiatric Symptoms in People Living with HIV Click here for additional data file. Supplemental material, sj-docx-1-try-10.1177_11786469221126888 for Microbiome-Related Indole and Serotonin Metabolites are Linked to Inflammation and Psychiatric Symptoms in People Living with HIV by Nadira Vadaq, Yue Zhang, Elise Meeder, Lisa Van de Wijer, Muhammad Hussein Gasem, Leo AB Joosten, Mihai G Netea, Quirijn de Mast, Vasiliki Matzaraki, Arnt Schellekens, Jingyuan Fu and André JAM van der Ven in International Journal of Tryptophan Research

sj-xlsx-2-try-10.1177_11786469221126888 – Supplemental material for Microbiome-Related Indole and Serotonin Metabolites are Linked to Inflammation and Psychiatric Symptoms in People Living with HIV Click here for additional data file. Supplemental material, sj-xlsx-2-try-10.1177_11786469221126888 for Microbiome-Related Indole and Serotonin Metabolites are Linked to Inflammation and Psychiatric Symptoms in People Living with HIV by Nadira Vadaq, Yue Zhang, Elise Meeder, Lisa Van de Wijer, Muhammad Hussein Gasem, Leo AB Joosten, Mihai G Netea, Quirijn de Mast, Vasiliki Matzaraki, Arnt Schellekens, Jingyuan Fu and André JAM van der Ven in International Journal of Tryptophan Research
