# Supplementary material for: Microbiome-Related Indole and Serotonin Metabolites are Linked to Inflammation and Psychiatric Symptoms in People Living with HIV
Source: Int J Tryptophan Res. 2022 Sep 27;15:11786469221126888. doi: 10.1177/11786469221126888 (PMC9520182; doi:10.1177/11786469221126888)
Supplement: sj-docx-1-try-10.1177_11786469221126888 – Supplemental material for Microbiome-Related Indole and Serotonin Metabolites are Linked to Inflammation and Psychiatric Symptoms in People Living with HIV [file sj-docx-1-try-10.1177_11786469221126888.docx]

**SUPPLEMENTAL MATERIALS**

**Microbiome-related indole and serotonin metabolites are linked to inflammation and psychiatric symptoms in people living with HIV**

Nadira Vadaq^1,2#^, Yue Zhang^3#^, Elise Meeder^4,5,6^, Lisa Van de Wijer^1^, Muhammad Hussein Gasem^2,7^, Leo A.B Joosten^1^, Mihai G. Netea^1,8^, Quirijn de Mast^1^, Vasiliki Matzaraki^1^, Arnt Schellekens^4,5,6^, Jingyuan Fu^3,9*^, André J.A.M van der Ven^1*^

1. Department of Internal Medicine, Radboudumc Center for Infectious Diseases, Radboud Institute of Health Science (RIHS), Radboud university medical center, Nijmegen, The Netherlands
2. Center for Tropical and Infectious Diseases (CENTRID), Faculty of Medicine, Diponegoro University, Dr. Kariadi Hospital, Semarang, Indonesia
3. Department of Genetics, University of Groningen, University Medical Center Groningen, Groningen, The Netherlands
4. Department of Psychiatry, Radboud University Medical Centre, Nijmegen, The Netherlands
5. Nijmegen Institute for Scientist-Practitioners in Addiction (NISPA), Nijmegen, The Netherlands
6. Donders Institute for Brain, Cognition and Behavior, Radboud University, Nijmegen, The Netherlands
7. Department of Internal Medicine, Faculty of Medicine Diponegoro University-Dr. Kariadi Hospital, Semarang, Indonesia
8. Department for Immunology and Metabolism, Life and Medical Sciences Institute, University of Bonn, Bonn, Germany
9. Department of Pediatrics, University of Groningen, University Medical Center Groningen, Groningen, The Netherlands

^#^These authors contributed equally to this work

*These authors contributed equally to this work and share the last authorship

Table of Contents

[Supplementary Figure 1. 3](#_Toc105938361)

[Supplementary Figure 2. 4](#_Toc105938362)

[Supplementary Methods 5](#_Toc105938363)

[Description of tryptophan metabolism pathways in PLHIV 5](#_Toc105938364)

[Association between gut microbial composition and tryptophan metabolism in PLHIV 5](#_Toc105938365)

[Comparison analysis of platelet serotonin concentration and association to platelet reactivity parameters 5](#_Toc105938366)

[Association between tryptophan metabolism and markers of inflammation and psychiatric symptoms in PLHIV 5](#_Toc105938367)

[References 6](#_Toc105938368)

## Supplementary Figure 1.


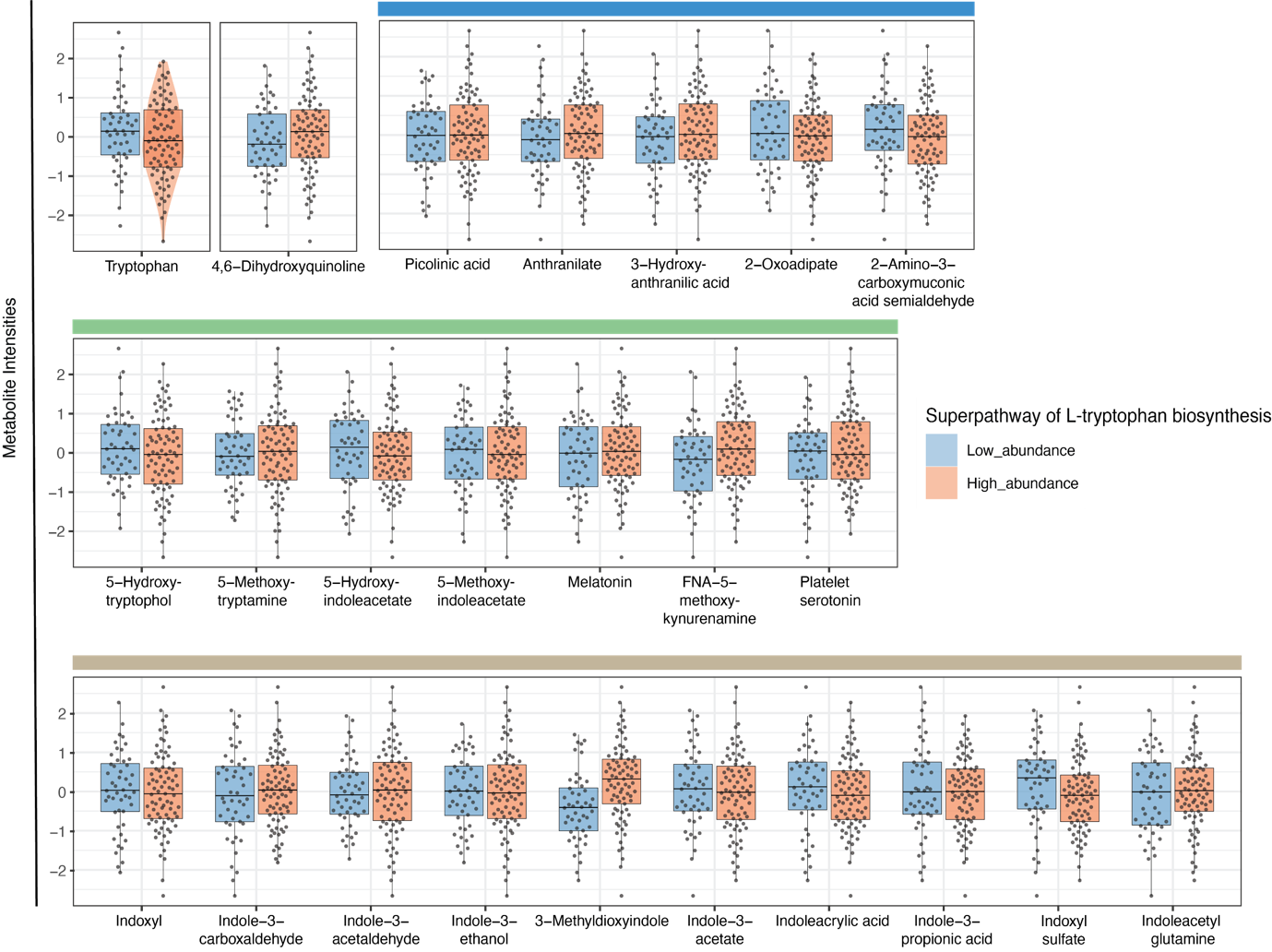


Supplementary Figure 3. Box plots depicting the intensities of tryptophan metabolism metabolites stratified by the high (n=81) and low abundance group (n=48) of “Superpathway L-Tryptophan biosynthesis” microbial pathway in PLHIV. In each box plot, the in-box line defines the median value, hinges depict 25th and 75th percentiles and whiskers extend to ±1.5 interquartile ranges; each dot indicates an individual participant. All values were inversed ranked based transformed. The analysis was performed using a linear regression model using age, sex, and number of reads.

Abbreviation: FNA-5-methoxykynurenamine = formyl-N-acetyl-5-methoxykynurenamine; 2A3-carboxymuconic acid acetaldehyde = 2-Amino-3-carboxymuconic acid acetaldehyde.

## Supplementary Figure 2.


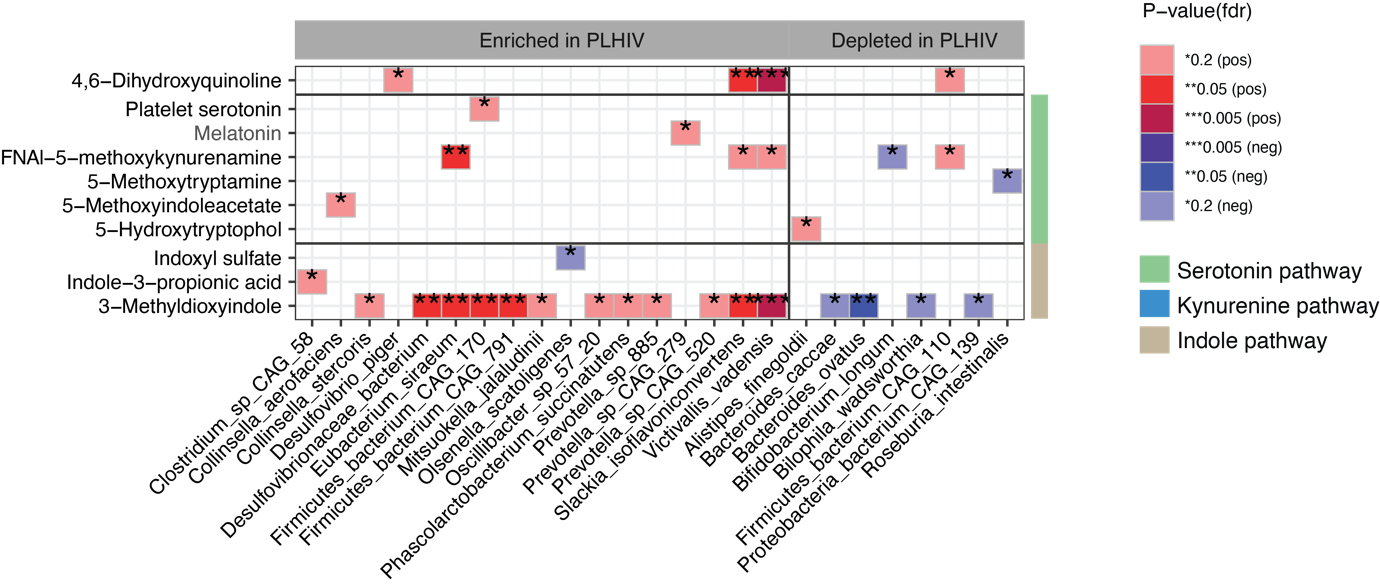


Supplementary Figure 3. Heatmap presenting significant associations (FDR<0.2) between tryptophan metabolism pathway and relative abundance of gut microbial species. The analysis was performed using a linear regression model using age, sexual orientation, and number of reads.

# Supplementary Methods

## Description of tryptophan metabolism pathways in PLHIV

The selection of metabolites belonging to tryptophan metabolism was performed using the Kyoto Encyclopedia of Genes and Genomes (KEGG) database ^1^ and the Human Metabolome Database (HMDB) ^2^. A total of 23 metabolites were identified (**Figure 1**), including tryptophan, 4,6-dihiydroxyquinoline, metabolites involved in serotonin pathway (melatonin, formyl-N-acetyl-5-methoxykynurenamine, 5-hydroxytryptophol, 5-methoxytryptamine,5-hydroxyindoleacetate, 5-methoxyindoleacetate), metabolites involved in kynurenine pathway (anthranilate, 3-Hydroxyanthranilic acid, 2-oxoadipate, 2-amino-3-carboxymuconic acid acetaldehyde, picolinic acid), and metabolites involved in indole pathway (indoxyl, indole-3-carboxaldehyde, indole-3-acetaldehyde, indole-3-ethanol, 3-methyldioxyindole, indole-3-acetate, indoleacrylic acid, indole-3-propionic acid, indoxyl sulfate, indoleacetyl glutamine). Details of selected metabolites are available in **Supplementary Table 2**. Serotonin and kynurenine were not measured in the assay as they were overshadowed by the nearest metabolites peak. Therefore, we measured the concentration of platelet serotonin separately. Platelet serotonin measurement was chosen because ~98% of peripheral serotonin is stored in platelet dense granules ^3^. Participants using selective serotonin reuptake inhibitors (SSRIs) were excluded from all the downstream analyses.

We first investigated the associations among metabolites from the tryptophan metabolism pathway in the 200 HIV cohort. Next, we assessed the activity of serotonin, kynurenine, and indole pathways using the composite values of the serotonin, kynurenine, and indole pathways. All associations were tested using Spearman’s rank correlation. The fixed composite value of serotonin, kynurenine, and indole pathways was calculated by a Z-scored transformation of a sum of downstream metabolites in each pathway divided by tryptophan concentration.

## Association between gut microbial composition and tryptophan metabolism in PLHIV

Microbiota compositions and differential abundance analysis have been described previously ^4^[preprint]. Briefly, we tested the differential abundance of bacterial species between PLHIV (200 HIV cohort) and HCs (DMP cohort) to identify bacterial species that enriched or depleted in PLHIV. Dysbiosis index score was then constructed by calculating the log2 ratio between geometric means of relative abundances of species that were enriched and depleted in PLHIV. Prior to association analysis, the relative abundance data were transformed using centered log-ratio transformation and bacteria detected in ≥20% samples were retained for downstream analysis (n bacterial species=99). We tested the association between tryptophan metabolism and the relative abundance of gut microbial species or dysbiosis index using a linear regression model adjusted for age, sex, and read counts.

## Comparison analysis of platelet serotonin concentration and association to platelet reactivity parameters

To compare the platelet serotonin concentration between PLHIV and HC, we used the measurements of PLHIV from the 200 HIV cohort and 56 healthy volunteers. To validate the results, we used measurements of PLHIV from a second independent PLHIV cohort (Art-NeCos) and 11 healthy volunteers. We used a linear regression model with adjustment for age and sex. Platelet serotonin concentration of participants using selective serotonin reuptake inhibitors (SSRIs) (eight subjects in the 200 HIV cohort and three in Art-NeCo cohort) were analyzed separately as SSRIs were known to block serotonin reuptake by serotonin transporter, resulting in intraplatelet serotonin depletion in platelet dense granules ^5^.

We next assessed the relationship between platelet serotonin concentration and platelet reactivity parameters in PLHIV using Spearman’s rank correlation. Prior to analysis, participants using platelet inhibitors (aspirin, clopidogrel, ticagrelor, and others) (n=23) were excluded.

## Association between tryptophan metabolism and markers of inflammation and psychiatric symptoms in PLHIV

For association analysis between metabolites from the tryptophan pathway and circulating inflammatory markers or psychiatric symptoms, we used a linear regression model with adjustment for age and sex.

# References

1. Kanehisa M, et al. KEGG: new perspectives on genomes, pathways, diseases and drugs. *Nucleic Acids Res*. Jan 4 2017;45(D1):D353-D361. doi:10.1093/nar/gkw1092

2. Wishart DS, et al. HMDB 4.0: the human metabolome database for 2018. *Nucleic Acids Res*. Jan 4 2018;46(D1):D608-d617. doi:10.1093/nar/gkx1089

3. El-Merahbi R, et al. The roles of peripheral serotonin in metabolic homeostasis. *FEBS Letters*. 2015;589(15):1728-1734. doi:<https://doi.org/10.1016/j.febslet.2015.05.054>

4. Zhang Y, et al. HIV-linked gut dysbiosis associates with cytokine production capacity in viral-suppressed people living with HIV. *bioRxiv*. 2022:2022.04.21.489050. doi:10.1101/2022.04.21.489050

5. Maurer-Spurej E, et al. The influence of selective serotonin reuptake inhibitors on human platelet serotonin. *Thromb Haemost*. Jan 2004;91(1):119-28. doi:10.1160/th03-05-0330
